# Comparison of radiographic and functional results of die-punch fracture of distal radius between volar locking plating (VLP) and external fixation (EF)

**DOI:** 10.1186/s13018-019-1442-0

**Published:** 2019-11-18

**Authors:** Yanqing Zhou, Yanbin Zhu, Xiong Zhang, Dehu Tian, Bing Zhang

**Affiliations:** 1 Department of Hand Surgery, The Third Hospital of Hebei Medical University, Shijiazhuang, Hebei 050051 People’s Republic of China; 2 Key Laboratory of Biomechanics of Hebei Province, Shijiazhuang, Hebei 050051 People’s Republic of China; 3 Department of Orthopaedic Trauma Center, The 3rd Hospital of Hebei Medical University, Shijiazhuang, Hebei 050051 People’s Republic of China

**Keywords:** Die-punch fracture, Distal radius, Radiographic and functional outcome, Volar plate fixation, External fixation

## Abstract

**Purpose:**

The aim of this study is to investigate the radiographic and functional results of die-punch fracture of distal radius treated by volar locking plate (VLP) or external fixation (EF).

**Methods:**

Between January 2015 and June 2018, 87 patients who were treated with EF or VLP were included in this study. At postoperative 6 months and at least 12 months, radiographic and functional outcomes were evaluated, and compared between two groups using SPSS 21.0.

**Results:**

The follow-up period was 15.6 months in average, and at the mean 8.5 weeks bony union was achieved in all patients. At 6-month visit, patients in VLP group had significantly better wrist flexion (79.2° vs. 71.8°) and pronation (79.5° vs. 75.2°) than those in EF group, but the difference was non-significant at the last visit (> 12 months); as for other parameters, no significant differences were observed. No significant difference was found between both groups in term of volar tilt, radial inclination, radial height, ulnar variance, or Gartland–Werley score and DASH. The articular step-off was significantly greater in EF than VLP group (0.6 mm vs. 0.3 mm, *p* < 0.001). The overall incidence of complications seemed higher in EF group (25% vs. 14%), but not approaching to the statistical significance level.

**Conclusions:**

Patients with VLP fixation of die-punch fractures had better wrist flexion and pronation at 6-month visit and more favorable wrist joint congruence at the last visit, but ultimately their outcome was comparable with those treated by EF.

## Introduction

Distal radius fracture is a very commonly seen injury, accounting for 7% to 25% of fractures in the populations of different age groups and 2.5% of all emergency department visits [1, 2]. In China alone, there were around 580,000 distal radius fractures during 2014 [3], and the incidence of distal radius fracture seems to be on the rise in China and abroad [4, 5].

Die-punch fracture of the distal radius was firstly described by Scheck in 1962 [6], as the dorsomedial fragments separated from the lunate fossa. Nowadays, this type of fracture is known as a special intra-articular fracture, mechanically a depression fracture of the lunate fossa caused by a vertical load through the lunate fossa. Although with surgical interventions, unfavorable outcomes have been widely reported, including increased rate of complications and poor functional recovery [7, 8]. We previously compared the outcomes of AO/ASIF type B distal radius fractures with and without lunate facet involvement treated by volar locking plate (VLP), and found the significantly higher incidence of articular step-off (19% vs. 4%) and poorer early-period outcomes (3 months) in the former one [7]. Likewise, Earp et al. [8] and Rozental et al. [9] found 50–63% of the postoperative reduction loss occurred in those of initial lunate facet involvement. Modification of the articular surface congruence of step-step over 2 mm could potentiate the risk of traumatic arthritis up to 78–100% [10, 11], which was associated with decreased motion range and loss of independence in daily activities.

Due to the unstable status, almost all the die-punch fractures necessitate the open reduction and locking plate/screws fixation via volar or dorsal approach. In addition, in some cases, percutaneous fixation with K-wires, mini-external fixator, non-locking plate/screws, wrist arthroscopy, intramedullary fixation, or combined use of two or more were used [7, 12, 13]. However, to our best knowledge, there is no consensus on the treatment of die-punch fractures, leading to the more difficulty in understanding of the functional results among the different surgical interventions for this type of injury.

This study aimed to compare the external fixator and the traditional VLP fixation for treatment of die-punch fractures, with regard to radiological parameters (volar tilt, radial inclination, articular step-off, ulnar variance), functional outcomes (wrist mobility, grip strength), and the overall functional assessment using disabilities of the arm, shoulder, and hand (DASH) and Gartland–Werley scoring systems.

## Material and methods

It was designed as a retrospective study and obtained approval by the ethics committee board of the 3rd Hospital of Hebei Medical University. All the patient had written informed consent at the beginning of the study. Between January 2015 and June 2018, patients diagnosed as die-punch fracture of the distal radius were included in this study. The inclusion criteria were age of 18 to 75 years; definite diagnosis of die-punch fracture of distal radius by preoperative radiographs or computed tomography scanning (CT) and reconstruction, if necessary; fresh fracture (< 21 days from fracture); no history of surgery at the injured hand; unilateral fracture and no concomitant fracture at the injured upper limb; surgical treatment by external fixator or VLP; and complete follow-up > 6 months. Patients with history of operation at the injured limb, existence of wrist osteoarthritis, lost to follow up, or unwilling to participate were excluded from the study.

A total of 87 patients met the criteria and included in this study. Fall from standing height was the most common cause in 32 patients, followed by motor vehicle collision (23), fall from greater height (14), sports injury (5) patients, machinery injury (3), and others (10). Sixty-seven patients had involvement of the right wrist and 20 patients had involvement of the left wrist. There were nine (10%) patients with concomitant ulnar styloid fracture.

The treatment decision for one patient was mainly made by his treating surgeon, in consideration of the surgeon’ experience, preference, reimbursement, and requirements of patients. There were 63 patients treated by VLP, including 25 men and 38 women, with average age of 52 years (SD, 15; range, 18 to 73 years). In external fixator group, there were 24 patients, including nine men and 15 women, with average age of 44 years (SD, 15; range, 18 to 66 years). Table 1 showed no significant difference between both groups in term of gender distribution, injury side, dominance, injury mechanism, and AO/ASIF classification. However, patients in the external fixation (EF) group were significantly younger (44 years vs. 52 years), and had a higher proportion of open fracture (25% vs. 3%).

**Table 1:** Demographic and injury-related data

	VLP group, *n* (%)	External fixation group, *n* (%)	*p*
Age (years)	52 (SD, 15)	44 (SD, 15)	< 0.001
Gender			0.852
Male	25 (39.7)	9 (37.5)	
Female	38 (60.3)	15 (62.5)	
Injured side			0.856
Left	7 (11.1)	3 (12.5)	
Right	56 (88.9)	21 (87.5)	
Dominant wrist fracture	58 (92.1)	22 (91.7)	0.952
Mechanism			0.256
Fall from standing height	25 (39.7)	7 (29.2)	
Motor vehicle collision	14 (22.2)	9 (37.5)	
Fall from higher height	11 (17.5)	3 (12.5)	
Sports injury	5 (7.9)	0	
Industrial machinery injury	1 (1.6)	2 (8.3)	
Others	7 (11.1)	3 (12.5)	
Type of fracture			0.002
Open	2 (3.2)	6 (25.0)	
Closed	61 (96.8)	18 (75.0)	
AO classification			0.681
Type B	24 (38.1)	8 (33.3)	
Type C	39 (61.9)	16 (66.7)	

### Surgical technique

#### VLP fixation

**Fig. 1:**
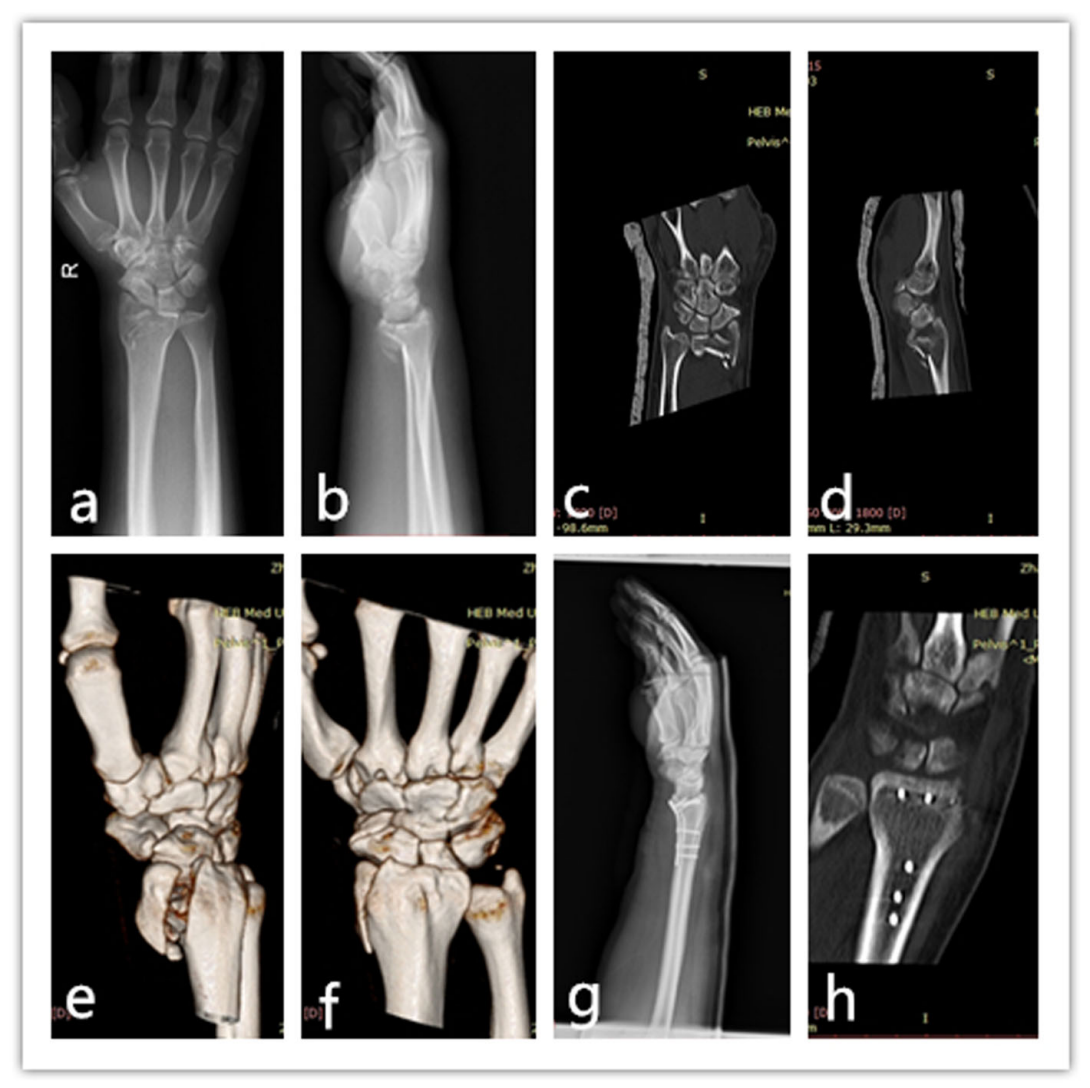
Patient, male, 39 years old, suffered from motor collision accident and sustained fracture in his left wrist. The preoperative radiographs (**a**, **b**) and CT scanning (**c**–**e**) showed the fracture of lunate facet with slight subsidence); and the fracture line extended to metaphysis. He underwent external fixation treatment (**f**–**h**) and the CT scanning showed the favorable wrist joint congruence

Classical Henry approach 10–12 cm in length was used. Flexor carpi radialis (FCR) and radial nerve and brachioradialis and radial blood vessels were retracted. Then, retract the pronator quadratus ulnarly and identify and reduced the fracture fragment under fluoroscopic guidance. As for transverse fracture line at the plane of impaction, periosteum elevator is introduced to elevate the fragments. For the longitudinal fracture line where fracture fragments are impacted longitudinally and separated from the lunate facet, periosteum elevator is introduced to disimpact the fracture fragments and then push them radially in line with the scaphoid facet (Fig. 1). Temporary fixation with Kirschner wires was used to stabilize the reduced fragments. After adequate reduction was achieved, T-shape locking plate (Synthes, Shanghai; Wego, Shandong, China) was applied and screws were placed, with auxiliary K-wires if necessary. Distal screws were placed just beneath the subchondral bone to provide the maximum ability to buttress the fracture fragments. The wound was then irrigated and closed. Postoperatively, cast immobilization was applied for 4 weeks. Under the surgeon guidance, early motion of finger, elbow, and shoulder was initiated on the first operative day, and wrist rehabilitation begun after cast and auxiliary K-wires removal after postoperative 4 to 6 weeks.

#### External fixation

**Fig. 2:**
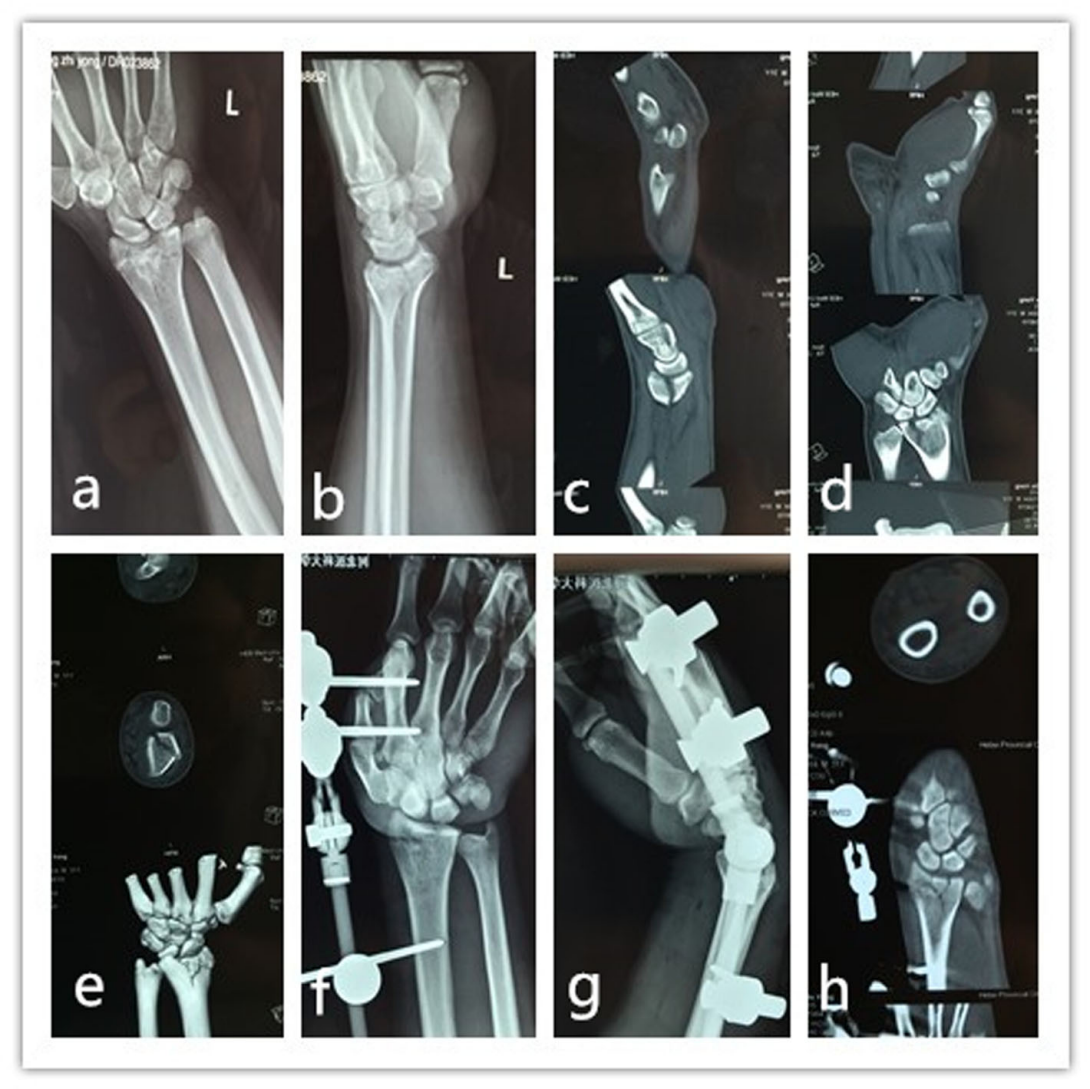
A male patient, 44 years old, sustained intra-articular fracture of right distal radius. In the outpatient, he was diagnosed as die-punch fracture based on radiographs (anteroposterior, **a** and lateral, **b**) and CT scanning (**c**–**f**). Finally, he was treated by VLP (**g**, **h**) and the CT scanning showed favorable reduction and fixation

For the die-punch fracture with slightly impacted fragments, continuous slight traction was performed to achieve and maintain the reduction under fluoroscopic guidance. Then, the external fixator (Stryker Trauma Corporation, Swiss) was directly fixed on the radius and the second or third metacarpal bone, with 4-mm Schanz pins and 3-mm pins applied respectively (Fig. 2). For fractures that were unsatisfactorily reduced or with the significant articular surface collapse or significant displacement of the larger fragment, a small incision was made at the volar side of the distal radius and the periosteum elevator was introduced to elevate the collapsed fragments. In cases of bone detect or seriously impacted fragments, allograft or allograft bone was implanted. After confirmation of the reduction under the fluoroscopic control, additional K wires were inserted for auxiliary fixation, if needed. All ulnar styloid fractures were not treated specially.

According to the literature reports [14, 15], satisfactory reduction was defined as dorsal tilt < 10°, volar tilt < 20°, radial inclination > 10°, radial shortening < 2 mm, and articular step-off < 1 mm at the immediate postoperative X-ray.

Under the guidance of surgeons, upper limb functional exercises were started at the postoperative 1–3 days. Three weeks, 6 weeks, and 3 months after surgery, regular X-ray check was performed and bone union status was evaluated. After 6 weeks, Kirschner wire and the external fixator were removed and wrist exercises were performed to increase the motion range of the operated wrist.

### Postoperative evaluation

The functional outcomes were wrist range of motion (ROM) and grip strength. ROM including wrist flexion, extension supination, and pronation was measured using a goniometer. Grip strength was measured using a Jamar dynamometer (Jamar, Preston, USA). All of these measurements were compared with that of the contralateral uninjured wrist. Subjective functional assessment was performed using the DASH score [16] and the Gartland–Werley scale [17]. The DASH is a validated patient-reported questionnaire that evaluates patients’ ability to perform the daily activities. The score range is 0 to 100, with 0 representing no disability and 100 points representing maximum disability. Gartland–Werley scale is a validated physician-based scoring system, combining residual deformity, subjective findings, the ROM, the postoperative complications, and poor finger function in consideration. The score ranges from 0 to 52 points, with higher score representing poorer result.

Regarding radiographic measurements as volar tilt, radial inclination, radial length and others, standard posteroanterior, and lateral radiographs were used. At the final radiographs, arthritic changes were judged using the Jupiter criteria [18]. At each visit, patients-reported complications or those from doctors’ examinations including infection, plate/screw loosening, neuropathy or nerve injury, tendon injury or tendinopathy, loss of reduction, chronic regional pain syndrome, and others were documented.

### Statistical analysis

The continuous variables, such as age, volar tilt, radial inclination, and others, were expressed as mean and standard deviation (SD). Student *t* test or Whitney *U* test was used to evaluate the difference, as appropriate. The categorical variables, such as gender distribution and complications, were expressed as number and percentage, and the difference between both groups was evaluated by Pearson chi-square test or Fisher’ exact test, as appropriate. The statistical test level was set as 0.05. SPSS 21.0 software (IBM, Armonk, NY, USA) was used to perform all the analyses.

## Results

The follow-up time was 15.6 months in average (12 to 42 months). At the mean 8.5 weeks (6 to 19 weeks), bony union was achieved in all patients.

Table 2 showed the results of wrist ROM and grip strength at the 6 months and the last visit (> 12 months). At the 6-month visit, VLP performed better than EF in term of wrist flexion (79.2° vs. 71.8°, *p* < 0.001) and forearm pronation (79.5° vs 75.2°, *p* = 0.036). However, at the last visit (> 12 months), the significance for these both parameters disappeared (*p* = 0.366, 0.848). Regarding other parameters such as extension, supination, radial deviation, ulnar deviation, and grip strength, no significant differences were observed at either time point (all *p* > 0.05).

**Table 2:** Comparison of ROM and grip strength of the operated wrist of the two groups at the 6-month and the last visit

	Six months				*p*	Last follow-up (> 12 months)				*p*
	VLP fixation		External fixation			VLP fixation		External fixation		
	Mean (SD)	% of value on contralat. side	Mean (SD)	% of value on contralat. side		Mean (SD)	% of value on contralat. side	Mean (SD)	% of value on contralat. side	
Flexion (°)	51.4 (10.5)	79.2	44.6 (11.5)	71.8	< 0.001	58.2 (9.6)	91.1	55.5 (9.2)	89.2	0.366
Extension (°)	54.6 (11.2)	80.2	52.4 (14.0)	77	0.176	61.6 (10.4)	92	60.8 (10.8)	92.4	0.927
Pronation (°)	79.5 (9.4)	88.4	75.2 (8.4)	85.1	0.036	81.2 (7.9)	93.6	79.7 (9.2)	91.8	0.848
Supination (°)	81.8 (8.4)	85.8	81.7 (7.4)	85.4	0.686	86.4 (8.8)	96.5	84.6 (10.6)	94.3	0.224
Radial deviation (°)	17.2 (6.6)	86.7	16.4 (7.5)	83.3	0.084	20.3 (5.8)	92.4	18.8 (7.4)	88.9	0.173
Ulnar deviation (°)	26.5 (8.4)	82.2	25.8 (9.3)	78.5	0.755	31.1 (5.3)	93.6	30.6 (4.8)	92.2	0.766
Grip strength (kg)	25.7 (7.7)	86.3	24.5 (8.5)	84.5	0.248	28.2 (9.4)	94.6	26.6 (7.9)	92.9	0.523

Regarding radiographic and functional results, no significant difference was found, in term of volar tilt, radial inclination, radial height, ulnar variance, or Gartland–Werley score (*p* > 0.05) (Table 3). Patients in VLP group seem to have a better DASH score (8.5 vs. 10.7), although the difference was marginally significant (*p* = 0.072). EF group had a less improved articular step-off than VLP group (0.6 mm vs. 0.3 mm, *p* < 0.001), indicating a poorer articular surface congruence.

**Table 3:** Comparison of radiographic parameters, functional outcomes, and complications between two groups at the last visit (> 12 months)

Variable	VLP	External fixation	*p*
Volar tilt (°)	5.7 (4.5)	5.3 (3.9)	0.528
Radial inclination (°)	22.6 (3.7)	21.4 (3.2)	0.318
Radial height (mm)	10.7 (1.2)	10.4 (1.4)	0.803
Ulnar variance (mm)	0.8 (1.1)	0.8 (0.9)	0.912
Articular step-off (mm)	0.3 (0.5)	0.6 (0.4)	0.003
DASH (points)	8.5 (9.7)	10.7 (12.0)	0.072
Gartland–Werley score (points)	4.9 (3.4)	5.3 (2.7)	0.173
Overall complications	9 (100)	6 (100)	0.237
Tendon complications	3 (37.5)	1 (20.0)	
Infection	0	2 (40.0)	
Traumatic osteoarthritis	0	1 (20.0)	
Neuropathy or nerve injury	1 (12.5)	1	
Hardware failure	1 (12.5)	0	
Complex regional pain syndrome Type I (CRPS)	2 (37.5)	1 (20.0)	
Carpal tunnel syndrome	2	0	

There were six (25%) complications in the EF group, and nine (14%) in the VLP group, but not different significantly (*p* = 0.341). In the EF group, there were two superficial pin tract infections which resolved with oral antibiotics; one patient developed a traumatic osteoarthritis (slight joint-space narrowing, grade 1, according to the system of Knirk and Jupiter). One patient in EF group and two patients in VLP group developed complex regional pain syndrome type I (CRPS), which required long-term physiotherapy. In VLP group, two patients had carpal tunnel syndrome, which resolved with immediate carpal tunnel decompression or long-term physiotherapy. Tendon complications including tendonitis or tendon contractures occurred in the VLP group (5%), but none in EF group.

## Discussion

In this study, we compared the functional and radiographic results of die-punch fractures of the distal radius treated by external fixator or VLP. We found that VLP performed better than EF in wrist flexion and pronation at the 6-month visit, and was associated with a favorable articular step-off at the final visit. As for other variables at 6-month or last visit, we found no significant differences. There were more complications in EF than in VLP group, but the difference did not approach to the statistical level.

It is of great importance to restore the congruence of the lunate facet, which is closely related to the wrist function recovery and prevention of traumatic osteoarthritis. The anatomic and radiographic studies showed that lunate facet accounted for 53% of the distal radius articular surface [19]. The three-column theory of wrist joint considered the lunate facet was the intermediate column, being the central movement zone for load bearing [20]. The relative ease of application and minimally invasive incision were the major advantages of external fixation, but the results might be partially compromised by the relatively poor reduction quality by ligamentotaxis. Especially in some cases where there is significant impaction between the metaphysis and cancellous bone or the lunate facet is split into volar or dorsal fragments, it is almost impossible to use ligamentotaxis even extreme traction to obtain satisfying reduction. In contrast, open reduction under direct vision and VLP fixation should be a better treatment choice. Besides, in VLP, the placement of screws as close to the subchondral line is very useful in buttressing small fragments and preventing radial shortening and articular displacement, especially for osteoporotic bone. Our finding of more favorable articular congruence in VLP group than EF group have demonstrated this.

Besides the manipulation of the fracture fragments under direct visualization, VLP fixation provides the possibility of immediate postoperative motion, which is associated with the better early-period wrist range of motion. In this study, we found that patients in VLP group had a better wrist flexion and forearm pronation at 6-month follow-up, which is consistent with a previous study of comparative results of external fixation versus VLP for treatment of unstable intra-articular distal radius fractures [13]. Relative to other fixation types, VLP also demonstrated the superior early recovery of wrist motion range [8, 9, 13]. However, the results of the two groups did not differ with regard to the DASH, Gartland–Werley score, wrist ROM, or radiographic parameters. It should be noted that, in our study, patients in EF group were older and had greater proportion of open fractures than VLP group. The relationship between age or open fracture and functional outcomes could not be identified in this study, but age had been identified as an independent factor that affected the wrist grip strength and wrist motion [21].

Complications after external or plate/screws internal fixation is always a problem, and the incidence was variedly reported. In a retrospective study of 206 cases of distal radius fractures by VLP, the overall incidence of complications was 10%, and incidence of tendon problems was 3% [22]. In a prospective study of 141 patients with unstable dorsally displaced distal radius fractures, authors reported the rate of overall complications was 27%, more being tendon problems (57%, 17/31) [12]. Authors suggested that this is attributed to the very distal placement of plates, the too long screws penetrating the extensor compartments, and the distal screws in comminuted fracture patterns causing screws to cut through and cause tendon irritation or rupture. In this study, we encountered only three cases of flexor tendon problems, but no extensor tendon problems, which might be related to increased knowledge anatomy of the distal radius and the awareness of surgeons in managing such injuries. The rate of pin track infection was 8% in EF in this study, consistent with the reported range [23]. By far, there was no sufficient evidences to support the hydroxyapatite coating of external fixator pins on pin tract infection [23], although some researchers advocated its use.

Carpal tunnel syndrome was more common in VLP fixation group, and their incidence rate could be up to 14% [24, 25], which occurred in the early or late postoperative period. In this study, two cases of carpal tunnel syndrome were found, one resolved with emergency decompression of the carpal tunnel with evacuation of hematoma and the other resolved with physiotherapy. Some researchers advocated the prophylactic transverse carpal ligament release to reduce such complications [26], but that was not routinely carried out in this study.

The limitations of our study should be mentioned. Firstly, this was designed as a retrospective, non-randomized study, which were associated with selection bias and data inaccuracy. Secondly, the procedures were performed by several surgeons and the choice of fixation type was mainly dependent on surgeons’ choice, not randomized and not blinded to evaluators. Thirdly, we were unable to assess the independent effect of some important variables on the functional results, due to the retrospective nature, such as the factors influencing the bone healing status [27], or the compliance of patients to perform the functional exercises. Fourthly, due to the small sample size, there is a possibility of type II statistical error. Future studies with large sample size and randomized and double-blind design are required to verify our results.

In conclusion, this study demonstrated the better performance of VLP fixation for die-punch fracture of the distal radius at early period (6 months) in term of wrist flexion and forearm pronation, compared to EF. At the last visit (> 12 months), VLP fixation was associated with better articular surface congruence, but not for other functional and radiographic parameters. There is a need for well-designed clinical studies with large size of participants to verify our results.

## Data Availability

Yes, data and meterial were available, not been published, and is not under consideration elsewhere.

## Abbreviations

AO: Arbeitsgemeinschaftfür Osteosynthesefragen
ASIF: The Association for the Study of Internal Fixation
CRPS: Complex regional pain syndrome
CT: Computed tomography
DASH: Disabilities of the arm, shoulder, and hand scale
EF: External fixation
FCR: Flexor carpi radialis
RCT: Randomized controlled trail
ROM: Range of motion
VLP: Volar locking plating
